# Is Erbium/Neodymium Laser Combination Therapy an Effective Treatment Option for Interstitial Cystitis/Bladder Pain Syndrome With Vulvodynia?

**DOI:** 10.7759/cureus.31228

**Published:** 2022-11-08

**Authors:** Nobuo Okui, Machiko Okui, Marco Gambacciani

**Affiliations:** 1 Dentistry, Kanagawa Dental University, Yokosuka, JPN; 2 Urogynecology, Yokosuka Urogynecology Clinic, Yokosuka, JPN; 3 Menopause and Osteoporosis, San Rossore Medical Center, Pisa, ITA

**Keywords:** estrogen therapy, vulvodynia, laser therapy, bladder pain syndrome, interstitial cystitis

## Abstract

Interstitial cystitis/bladder pain syndrome (IC/BPS) is often associated with vulvodynia and poor vaginal health. IC/BPS causes pelvic and bladder pain and urinary symptoms, which considerably reduce the quality of life. To date, this condition has no definitive cure. Local estrogen therapy (LET) has been proposed as a treatment for vulvodynia and poor vaginal health to improve the symptoms of IC/BPS. However, chronic LET could be contraindicated or not desired in some patients. The present study reports the case of a 55-year-old postmenopausal woman with IC/BPS who was successfully treated with combined vaginal erbium (VEL)/neodymium (Nd:YAG) laser (VEL+Nd:YAG) therapy. The patient presented with a five-year history of pelvic pain and urinary frequency. Direct approaches for the bladder (such as hydrodistension, anticholinergic drugs, and transurethral Hunner lesion ablation/cauterization) were conducted with inconsistent results. Immediately prior to the patient’s presentation, LET was administered for 12 weeks; however, this therapy resulted in mild improvement and poor patient satisfaction. After presentation, VEL+Nd:YAG therapy was conducted once a month for three months. The patient reported considerable decrease in pain during urination. The improved symptoms were maintained for six months after the last therapy session. These results suggest that VEL+Nd:YAG therapy is an effective method for improving symptoms in patients with IC/BPS.

## Introduction

Interstitial cystitis/bladder pain syndrome (IC/BPS) comprises persistent or recurrent chronic pelvic pain and bladder-associated discomfort. IC/BPS is accompanied by at least one urinary symptom, such as urinary urgency or urinary frequency, and is diagnosed with the exclusion of other identifiable medical conditions that can explain the patient’s symptoms. The etiology of IC/BPS is unclear [1,2]. In the United States, the prevalence of IC/BPS is 2.7-6.5% among women. Various treatment methods have been proposed, and the definition and etiology of IC/BPS are controversial [2]. However, no definitive treatment is widely accepted.

Analgesics, lifestyle changes, and patient education are recommended as first-line treatments for IC/BPS. Intravesical therapy (dimethyl sulfoxide (DMSO), lidocaine, or heparin) and oral therapies, such as amitriptyline, are recommended as second-line methods. Invasive surgical treatments include cystoscopic hydrodistension, transurethral Hunner lesion ablation, and cystectomy [1]. IC/BPS is often accompanied by vulvodynia and poor vaginal health [3,4]. Therefore, treatments for vulvodynia and poor vaginal health have been proposed for IC/BPS treatment [5]. Reportedly, vulvar and bladder pain improved after 12 weeks of local estrogen therapy (LET) within the vagina and vaginal vestibule, suggesting an association between vulvar pain, bladder pain, and vaginal health [5]. However, many women do not wish to undergo long-term daily LET, and hormone therapy is contraindicated in patients with a history of estrogen-dependent tumors, such as breast cancer.

Combined vaginal erbium (VEL)/neodymium (Nd:YAG) laser (VEL+Nd:YAG) therapy is a proposed alternative to LET for vulvar pain [6]. It involves the simultaneous implementation of VEL:YAG laser irradiation accompanied by long pulse and very weak vulvar Nd:YAG laser irradiation. In superficial dyspareunia, VEL+Nd:YAG therapy is significantly more effective for improving vulvar pain than VEL alone [6]. Improvements in urinary symptoms were reported in women with IC/BPS who underwent monthly VEL for one year [7]. We previously reported the use of vaginal energy-based devices (EBDs) in IC/BPS patients for the first time [8]. VEL is considered a local therapy, conducted around rather than within the bladder.

Here, we report the case of a patient with refractory interstitial cystitis and vulvodynia treated with VEL+Nd:YAG therapy directed at the vagina and vulva.

## Case presentation

A healthy, 55-year-old woman (gravida 3 para 1; body mass index: 26.2 kg/m^2^) presented with a five-year history of urinary frequency and pelvic area pain. She regularly experienced severe pain in the upper pubis throughout the day. Previous treatments, including lifestyle changes, various analgesic therapies, oral hydroxyzine therapy, anticholinergic therapy, selective β3 adrenaline receptor agonist therapy, intravesical DMSO injection, and transurethral Hunner lesion ablation/cauterization, were ultimately ineffective. The patient was not receiving any anti-psychotic drugs such as amitriptyline hydrochloride, had no history of pelvic surgery or photosensitivity, and underwent menopause at the age of 49.

At a previous hospital, the patient underwent cystoscopic hydrodistension under general anesthesia. The bladder had dilated capillaries with hydrodistension at rest. Physiological saline was removed after three minutes, and post-distension mucosal hemorrhage, glomerular formation, and partial Hunner lesions were noted. Accordingly, the patient was diagnosed with IC/BPS. Her pain returned three months after intravesical water pressure-based dilation.

Immediately before presentation, the patient underwent transvaginal LET with estriol 0.5 mg (estradiol dipropionate, ASKA Pharmaceutical Co., Ltd. Tokyo, Japan). She reported mild improvements and overall dissatisfaction with the outcomes. Six months after the last treatment at the previous clinic, she visited our outpatient office. Upon presentation, her pelvic pain and urgency/frequency (PUF) symptom score was 22. The PUF includes 12 items divided into two domains: symptom and bother scores. The total PUF score ranges from 35 (maximum; severe problems) to 0 (minimum; no problems). No cystocele, rectocele, or descensus was detected, and stress tests showed negative findings. Further, the Pap smear, urinalysis, urine culture, and urine cytopathology findings were negative. Based on vulvar atrophy and poor vaginal health, the patient was diagnosed with genitourinary syndrome of menopause. The patient’s Vaginal Health Index Score (VHIS) was 10 (total VHIS range: 25 (healthy) to 5 (poor)) [3,4].

The patient reported vulvar spontaneous pain, and vulvar swab tests produced vulvar provoked pain, especially around the urethra. In the uroflowmetry study, her maximum urine flow rate (Qmax) was 35 mL/s and voided volume (VV) was 110 mL, with no residual urine detected. Her three-day urinary diary revealed that she urinated 40 times/day. Urination frequency was high when many genital and urethra stimulating activities were performed, including sitting on a chair, riding a train, and doing desk work (Table 1). In the cystometric study, the bladder was completely emptied using a catheter. A small, special catheter, which had a pressure-measuring manometer, was inserted into the bladder, and warm water slowly filled the bladder. Our patient reported the first desire (FD), normal desire (ND), and maximum capacity (MCC) at 72.3 ml, 97.9 ml, and 110 ml, respectively (Table 2). The patient complained of pain in the urethra, which persisted throughout the examination, when the catheter was inserted. During the cystometry, at MCC, she complained of severe pain in the bladder. At the start of infusion, vesical pressure (Pves) and urethral pressure (Pura) were 31.8 and 21.1 cm H_2_O, respectively. The urethra was painful, but there was no strong sphincter or obstruction of the urethra. Based on the volume of water infused (VH_2_O) and Pves at FD, ND, and MCC, the patient was not diagnosed with low-compliance bladder, which is defined as a significant increase in bladder pressure and a slight increase in bladder volume. In addition, despite urethral pain, detrusor overactivity was not observed. The Goby Family of Wireless Urodynamics Systems (EDAP TMS SA, Rhône, France) was used in the uroflowmetry and cystometric studies.

**Table 1:** Changes in the urination diary before and after laser treatment Typical records are shown. The patient works from 9:00 am to 5:00 pm.

T0			T3			T6		
Time	Drink (ml)	Urination (ml)	Time	Drink (ml)	Urination (ml)	Time	Drink (ml)	Urination (ml)
7:00 AM	200	120	7:00 AM	250		7:00 AM	300	
8:00 AM	150	75	8:00 AM	150		8:00 AM	150	
8:15 AM		5	8:35 AM		100	8:30 AM		100
8:35 AM		45	9:00 AM	120		9:00 AM	100	
9:00 AM	100	80	11:30AM		300	11:30AM		230
9:15AM		15	12:10 PM	250		12:10 PM	250	
9:30AM		10	12:40PM		50	1:00PM	200	250
10:15 AM		75	1:20PM	200		3:00 PM	200	100
10:20AM		10	1:40PM		250	5:00 PM	100	
11:00 AM		75	3:00 PM	200		5:10PM		300
11:30AM		90	3:55PM	100		6:00 PM	100	
12:10 PM	250	40	5:00 PM	100		8:00 PM	200	
12:40PM		130	5:10PM		300	10:15PM		200
1:00 PM	150	100	6:00 PM		200	12:00 AM		250
1:20PM		5	8:00 PM	200		6:00 AM		350
1:40PM		50	9:00 PM		100			
2:00 PM		20	10:15PM		80			
3:00 PM	200	75	12:00 AM		250			
3:30PM		25	6:00 AM		310			
3:48PM		30						
3:55PM		5						
4:20 PM	120	10						
5:00 PM	100	100						
5:10PM		15						
5:20PM		20						
5:40PM		5						
5:55PM		5						
6:00 PM		5						
6:50PM		15						
7:50 PM		70						
8:00 PM	200	5						
9:00 PM		100						
9:50PM		20						
10:00 PM		5						
11:00 PM		5						
11:50PM		20						
1:00 AM		120						
3:00 AM		150						

**Table 2:** Changes in pain and urination symptoms before and after laser treatment FD, ND, and MCC show VH_2_O (ml) and Pves (cm H_2_0). Pves are provided within brackets. PUF: total pelvic pain and urgency/frequency symptom score; VHIS: total vaginal health index score; Swab tests: vulvar pain and vulvar swab tests; UF: urinary frequency per day from urinary diary; Qmax: maximum urine flow rate; VV: voided volume; FD: first desire; ND: normal desire; MCC: maximum capacity; VH_2_O: volume of water infused; Pves: vesical pressure

Examination	T0	T3	T6
PUF	22	4	4
VHIS	10	23	25
Swab tests	Provoked pain, especially around the urethra	No pain	No pain
UF	40	10	8
Qmax	35 mL/s	34 mL/s	35 mL/s
VV	110 mL	310 mL	350 mL
FD	72.3 ml (31.7 cm H_2_O)	190 ml (30.1 cm H_2_O)	213 ml (29.9 cm H_2_O)
ND	97.9 ml (33.8 cm H_2_O)	220 ml (30.4 cm H_2_O)	287 ml (29.4 cm H_2_O)
MCC	110 ml (36.2 cm H_2_O)	310 ml (70.2 cm H_2_O)	350 ml (73.1 cm H_2_O)

**Figure 1:**
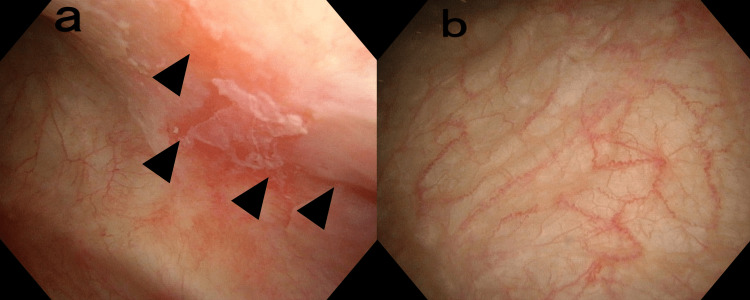
Cystoscopic changes before and after laser treatment Inflammation and Hunner lesions (arrows) were observed before laser treatment (T0); these were not observed six months after the last laser treatment session (T6).

**Figure 2:**
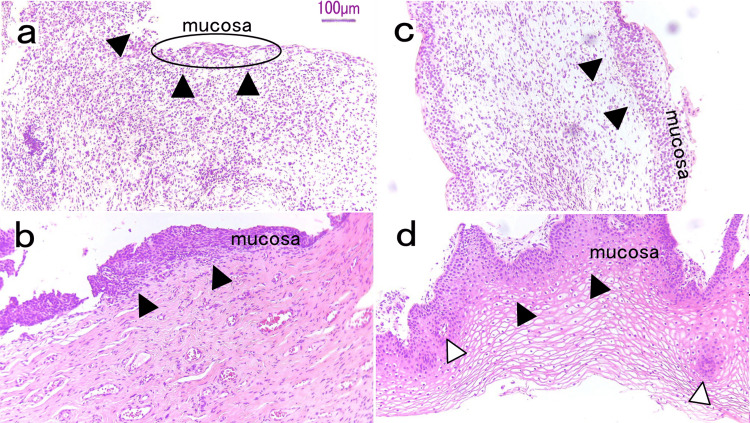
Histological changes before and after laser treatment Bladder biopsies were performed before laser treatment (T0) and six months after the last laser treatment session (T6). The specimens were fixed in formalin and embedded in paraffin. Sections were stained with hematoxylin and eosin for an overview of the mucosal tissue. Microscopic observations were conducted using standard light microscopy (Primo Star, Carl Zeiss, Germany) at 4× magnification. (a) At T0, the bladder biopsy revealed severe inflammation. The mucous membrane was detached except for small mucous membrane layers (ellipse). Several inflammatory cells were observed (arrows); (b) At T0, the vaginal mucosal tissue was thin (arrows); (c) At T6, improvement in stromal inflammation was seen. The bladder mucous membrane was regenerated and had a normal thickness (arrow marks); (d) At T6, the vaginal mucosal tissue showed significant improvement. A thickened mucous membrane was observed (black arrows), and the blood vessels were regenerated (white arrows).

Cystoscopy confirmed hydrodistension at rest, and a random bladder biopsy revealed exfoliated epithelium with erosions, proliferative blood vessels, and conglomerations of submucosal inflammatory cells, diagnosed as severe stromal inflammation and Hunner lesions (Figures 1a, 2a). The vaginal mucosal tissue was thin with few epithelial cells and blood vessels (Figure 2b). After the random bladder biopsy (T0), the patient chose to undergo three laser irradiations (L1, L2, and L3) conducted once a month over three months after counseling and approval from the Institutional Review Board (approval number: IRB1900-5) with written informed consent. Follow-up was conducted three (T3) and six (T6) months after the final laser irradiation session. Bladder endoscopy and vaginal mucosal biopsies were conducted at T0 and T6 to confirm the therapeutic effects of VEL+Nd:YAG and rule out malignant tumors as no cases of VEL+Nd:YAG therapy for the treatment of IC/BPS have been reported. Bladder and vaginal epithelium thicknesses were measured at six random locations, and the average was recorded [9].

**Figure 3:**
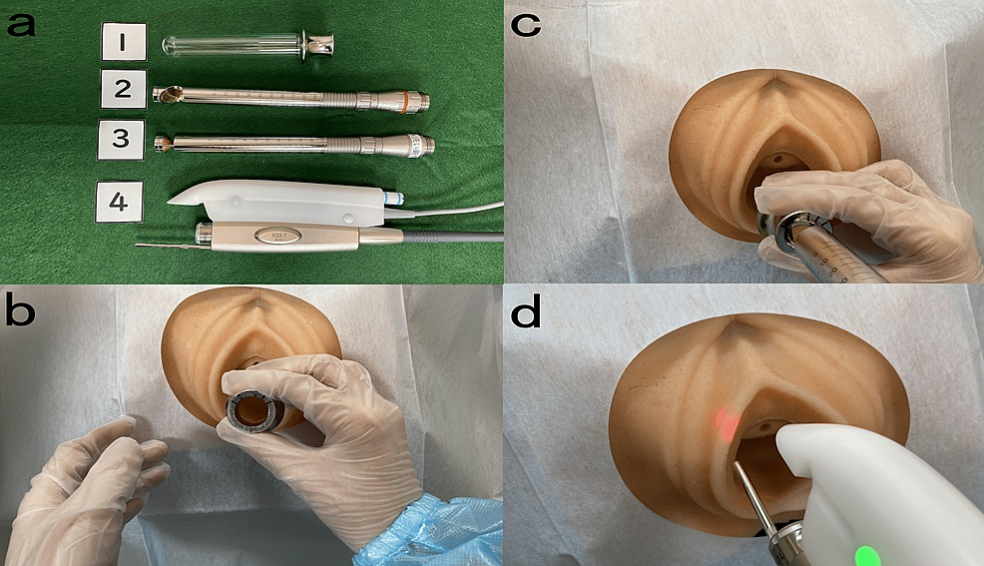
Detailed steps of the procedure (a) All handpieces required for this procedure. The MClear Speculum (1) is a transparent, reusable (after sterilization) vaginal speculum. PS03 (2) and R11(3) handpieces are angular + circular golden-mirror adapters. The R33 Nd:YAG (4) handpiece has a variable flat-beam spot size from 2 to 10 mm. (b) Vaginal erbium laser (VEL) therapy. The MClear Speculum is inserted into the vagina. (c) R11 with circular GC adapter is inserted into MClear Speculum and the laser irradiation is delivered at every 5 mm of vaginal canal length to achieve circular therapy of the whole vaginal wall. Then, the R11 and speculum are removed, and PS03 with a spot size of 7 mm is used for irradiation of the vestibule and introitus. (d) Nd:YAG therapy. After completing the VEL step, six passes were conducted in the brushing mode to treat the posterior commissure with The R33 Nd:YAG handpiece. VEL: vaginal erbium; Nd:YAG: neodymium

VEL+Nd:YAG therapy was conducted at an outpatient clinic (Figure 3). Before the procedure, the patient’s vagina was washed with an antiseptic solution and dried with a cotton swab. Renovalase^TM^ (Fotona SP Dynamis, SMOOTH mode; Fotona, Ljubljana, Slovenia) protocol was used at a wavelength of 2,940 nm. The laser spot diameter was 7 mm, frequency was 1.6 Hz, and pulse fluence (laser energy emitted per unit area) was 1.75 J/cm^2^, as previously reported, to prevent tissue ablation with a deep thermal effect [6,7]. A specially designed glass vaginal speculum was used to insert the probe (R11) into the vaginal speculum and avoid contact with the vaginal mucosa. After VEL therapy, Nd:YAG therapy was conducted using an Nd:YAG laser (Fotona SP Dynamis, PIANO mode), an R33 non-contact handpiece with a spot size of 9 mm, PIANO pulse mode (5 s), and fluence of 90 J/cm^2^. Six passes were conducted in the brushing mode to treat the posterior commissure [6]. The VEL and Nd:YAG therapies were conducted during the same sessions with no gap between exposures to the two energies. The patient was advised to avoid sexual activities for one week following each session.

The patient’s symptoms were alleviated at each follow-up. The urination interval at T3 was longer than that at T0, and the urinary frequency decreased from 40 times/day at T0 to 10 times/day at T3 and eight times/day at T6, with a similar daily fluid intake (Table 1). The patient reported considerably decreased pain during urination, and the PUF score improved to 4 at T3, which was maintained at T6 (Table 2). The patient reported improved symptoms at T6. The VHIS increased from 10 to 23 at T3 and 25 at T6 (Table 2). In the uroflowmetry study, the Qmax showed no change, but the VV improved. In the cystometric study, pain in the urethra caused by the examination catheter disappeared after treatment. The FD, ND, and MCC were improved, and the pain at MCC was particularly reduced (Table 2). The vulvar swab test was negative. At T6, there were no Hunner lesions (Figure 1b). A random bladder biopsy revealed significantly improved stromal inflammation, with no inflammatory cells (Figure 2c). In the vaginal mucosa, the glycemic content was significantly increased with more epithelial cells, new papillae, and angiogenesis that supported a new lamina propria and reached the epithelium (Figure 2d). In the bladder, the average thickness of the epithelium before and after treatment were 8.33±12.1 µm and 106.7±35.9 µm, respectively. In the vaginal wall, before treatment, the average thickness was 57.3±14.2 µm, while after treatment it was 133.3±24.5 µm.

## Discussion

To the best of our knowledge, this is the first report of improved vaginal, vulvar, and bladder pain in a patient with IC/BPS treated with VEL+Nd:YAG therapy. VEL+Nd:YAG therapy can be considered a minimally invasive therapy conducted around the bladder, whereas conventional surgical therapies for IC/BPS are performed inside the bladder.

Women with IC/BPS are thought to be vulnerable to sex hormone-related changes, even if IC/BPS occurs before menopause. Patients with IC/BPS are susceptible to abnormal reactions of the genital tissue due to genetic and/or acquired factors. LET is reported to be effective for patients with IC/BPS with vulvodynia and poor vaginal health [5]. The efficacy of LET may be because the vulva and bladder share innervation due to their same embryonic origin [10]. In addition, estrogen has been reported to improve nerve density and fiber type [11]. The potential regulatory role of estrogen regarding pain thresholds, neuronal inflammation, and immune response may have genomic and non-genomic effects [12]. However, LET resulted in mild, unsatisfactory improvement in our patient, which may be due to the severe atrophy of the vagina and vulva detected at T0. The PUF scores revealed pain levels in our patient to be worse than that generally noted in patients with IC/BPS, with the mean total PUF score previously reported as 18.19±3.52 in patients with IC/BPS and 3.76±2.35 in healthy women [3]. Similarly, our patient also had vulvodynia and poor vaginal health. Her VHIS score was worse than the average score of patients with IC/BPS, with the mean total VHIS score previously reported as 12.03±3.36 in patients with IC/BPS and 22.05±3.90 in healthy women in Asia [3].

The effectiveness of VEL+Nd:YAG therapy may be independent of years since menopause as it can be conducted in post-menopausal women with superficial dyspareunia [6]. VEL therapy involves energy carried by laser irradiation that reacts with the moisture in the mucous membrane to generate heat, achieving a resurfacing effect. VEL therapy effectively alters the vaginal epithelium in patients with severe vaginal atrophy [9], resulting in the recovery of the epithelium of the vagina and urethral mucosa, increased epithelial metabolic cycling, glycogenic loads, papillomatosis, and collagen formation, and enlarged cellularity of the lamina propria. A study on 35 women reported that micro-ablation Er:YAG laser therapy significantly improved the symptoms of vulvodynia, but not more than that achieved with other multidisciplinary therapies [13]. VEL therapy differs from Er:YAG laser therapy in terms of the micro-ablative mode; it is non-ablative and delivers controlled warming and fast heat shock into the target tissue [14]. A previous case report showed non-ablative Er:YAG laser therapy to be effective for vulvodynia [15]. Moreover, 28 patients with a clinically conﬁrmed diagnosis of vulvar lichen sclerosus experienced significant improvement after VEL therapy [16]. Good efficacy has also been reported for VEL therapy in women with IC/BPS, but these effects were only observed after VEL was performed every month for a year [7].

In contrast to VEL therapy, Nd:YAG laser therapy is effective for uniform bulk heating of tissues, such as the dermis and subcutaneous tissue, and very deep structures can be reached with a longer pulse width, such as that used in the PIANO mode in this report. In a pilot study, Gambacciani and Fidecicchi [6] showed that VEL therapy alone significantly improves superficial dyspareunia, but that the combination of VEL and PIANO Nd:YAG has much better results. Patients with lichen sclerosus who underwent PIANO Nd:YAG therapy reported significantly improved symptoms (such as burning sensation, itchiness, pain, and dyspareunia) and fewer skin inflammatory cells than patients treated with topical corticosteroids [17]. As already mentioned, given the relationship between the external genitalia and bladder, there may be some interaction between the vagina and bladder, represented by estrogen. Although VEL therapy improves bladder symptoms, it requires multiple VEL sessions for a long period. Targeting the genitals as well as the vagina reduces the number of treatment sessions. Moreover, the effect on the bladder was conspicuous. Normal Qmax and VV values vary widely in the literature; Qmax is >30 mL/s and VV is 200-250 mL in healthy women. Qmax data showed that the detrusor muscle was not affected either before or after treatment. VV data showed that our patient had abnormal urine volume owing to pain, but the volume improved and was normal after treatment. In healthy women, the reference range for urinary frequency is 2-10 times per day and zero to four times per night [18]. The urinary frequency in our patient normalized after treatment, which we believe is related to pain improvement.

Hyperbaric oxygen therapy (HBO) [19] and transvaginal photobiomodulation (TV-PBM) are used for minimally invasive therapy around the bladder [20]. HBO involves oxygen inhalation at a higher concentration than that in air, above atmospheric pressure. Seven of 11 patients with IC/BPS resistant to conventional therapy who received 10 or 20 HBO treatments showed good improvement for at least one year. However, HBO requires a large equipment room [19]. Butrick et al. reported EBD treatment for IC/BPS patients in 2022 [20]. Their idea conforms with our previous report in 2020 [7]. TV-PBM uses near-infrared (NIR) light to target the mitochondrial chromophore cytochrome c oxidase (COX). NIR activation of COX initiates intracellular chemical reactions leading to analgesia, improved oxygenation, and reduced inflammation, edema, and muscle relaxation. Of 140 patients with IC/BPS, two-thirds reported significant reductions in pelvic pain and dysuria after four to eight treatments [20]. While these two reports did not confirm the treatment effects in vulvar swab test and VHIS, they demonstrated the importance of therapy conducted around the bladder, similar to our case report.

In this report, histological improvements in vulvar and vaginal health were observed after laser therapy was conducted around the bladder, leading to improved symptoms of IC/BPS. VEL+Nd:YAG therapy may be effective for patients with poor vaginal health and vulvodynia, which are often complicated by IC/BPS, and the effects are maintained for up to six months after treatment.

## Conclusions

Here, we described the effects of VEL+Nd:YAG therapy in a woman with IC/BPS who did not improve even after multiple conventional treatments. Nevertheless, this treatment resulted in improved vaginal and vulvar symptoms, as well as bladder symptoms. VEL+Nd:YAG therapy can be a potential alternative therapy for patients with resistant IC/BPS. Our report and literature review demonstrate the importance of evaluation of vaginal impairment and vulvodynia and that of a minimally invasive therapy conducted around the bladder. Further randomized controlled trials and prospective studies are needed to elucidate the etiology and mechanisms of IC/BPS.
